# Supplementary material for: Exploiting pyocyanin to treat mitochondrial disease due to respiratory complex III dysfunction
Source: Nat Commun. 2021 Apr 8;12:2103. doi: 10.1038/s41467-021-22062-x (PMC8032734; doi:10.1038/s41467-021-22062-x)
Supplement: Supplementary file 1 — Supplementary Information [file 41467_2021_22062_MOESM1_ESM.pdf]

Figure S1

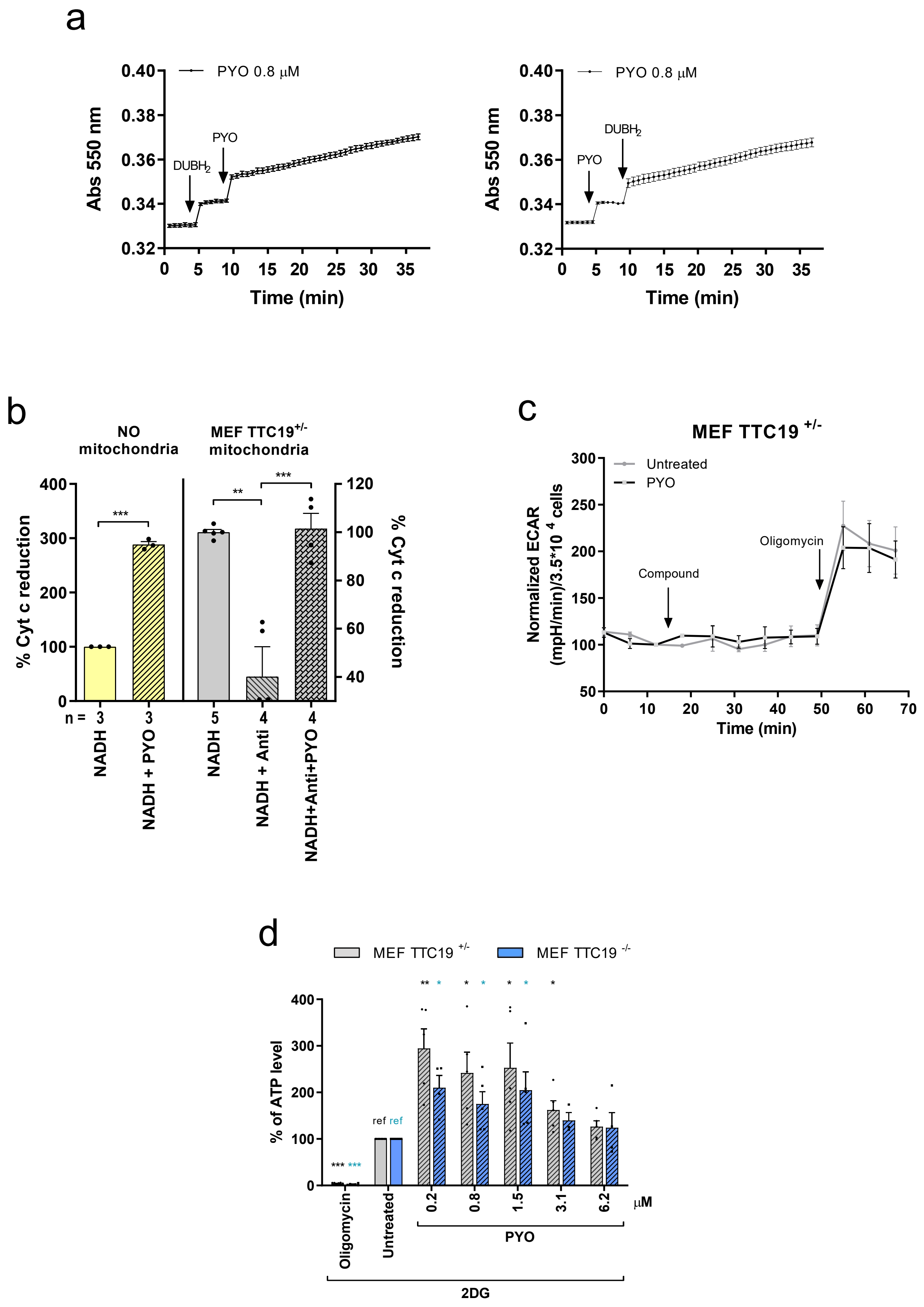
a
PYO 0.8 μM
Abs 550 nm
Time (min)
DUBH2
PYO
b
NO mitochondria
MEF TTC19+/- mitochondria
% Cyt c reduction
n = 3 3 5 4 4
NADH
NADH + PYO
NADH
NADH + Anti
NADH+Anti+PYO
c
MEF TTC19 +/-
Untreated
PYO
Normalized ECAR (mpH/min)/3.5*10 4 cells
Compound
Oligomycin
Time (min)
d
MEF TTC19 +/-
MEF TTC19 -/-
% of ATP level
ref ref
Oligomycin
Untreated
0.2
0.8
1.5
3.1
6.2
μM
PYO
2DG

SUPPLEMENTARY FIGURE LEGENDS

Figure S1. **PYO affects bioenergetics in MEF cells.**

a) PYO accepts electrons from decylubiquinol and donates them to cytochrome c in the absence of mitochondria. Experiments were performed as described in the Methods section, without mitochondria. Compounds were added as indicated (means ± SEM, n=4 independent samples). **b)** CIII activity was measured monitoring cytochrome c reduction as described in the Methods section. The capability of PYO to accept electrons from NADH was tested both in the absence of mitochondria, and in isolated mitochondria from MEF TTC19$^{+/-}$ after CIII inhibition by antimycinA (means ± SEM). The number of measures over 3 independent samples is shown in the image. **c)** Extracellular Acidification Rate (ECAR) was measured in MEF TTC19$^{+/-}$ after the addition of 1.5 µM PYO (means ± SEM, n=3 independent experiments). **d)** Experiment performed as in Figure 1l but the data are reported as percentage of the luciferase signal emitted by the untreated sample and specified in the graph as "ref" (means ± SEM, n=5 (MEF TTC19$^{+/-}$) or 6 (MEF TTC19$^{-/-}$) independent experiments). For all the panels statistical significance (one-way ANOVA with Dunnett's Multiple comparison test or two-tailed Student's *t*-test analysis) was determined (* = p<0.05; **=p<0.01; ***=p<0.001).

Figure S2

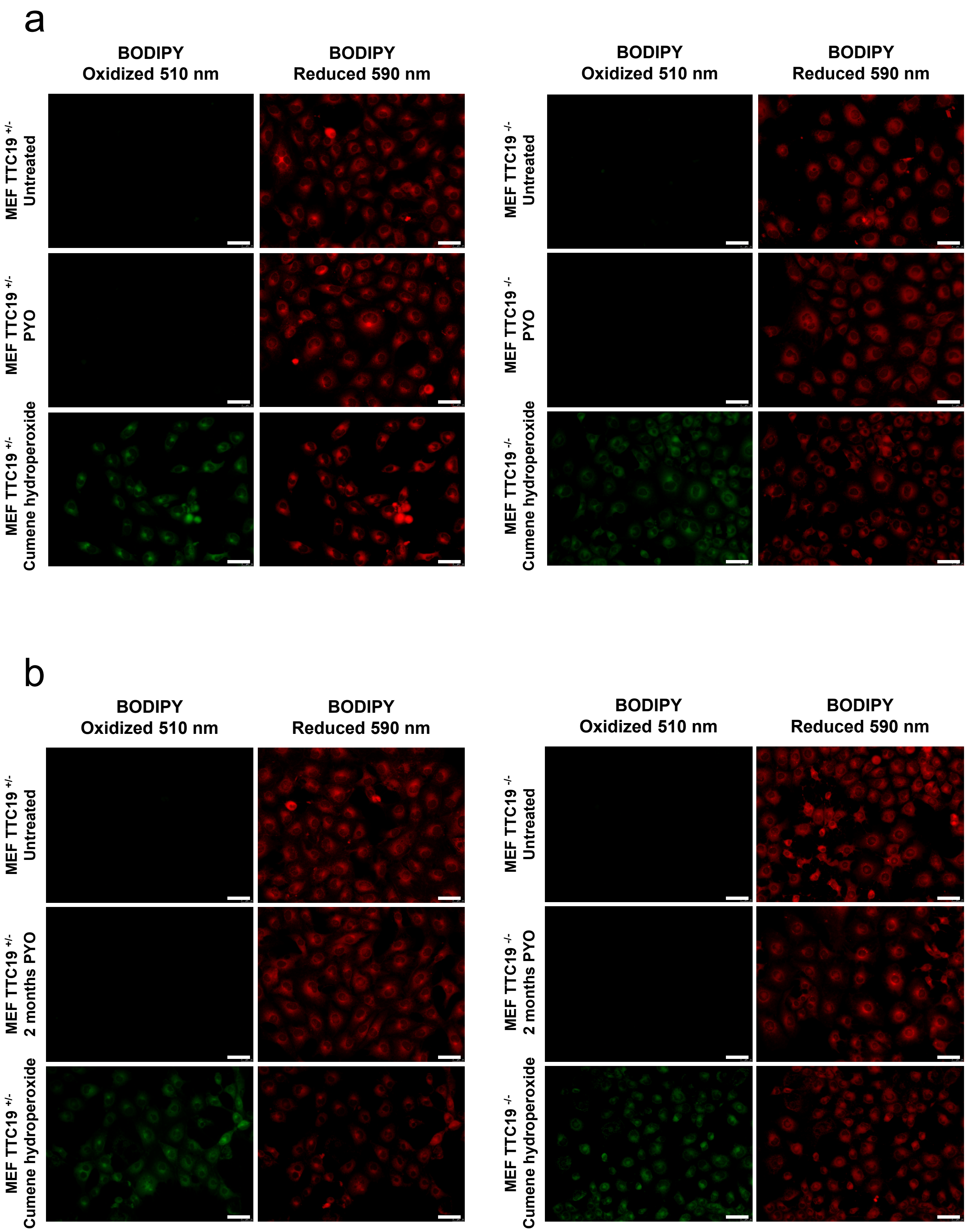
a
BODIPY Oxidized 510 nm
BODIPY Reduced 590 nm
BODIPY Oxidized 510 nm
BODIPY Reduced 590 nm
MEF TTC19 +/- Untreated
MEF TTC19 +/- PYO
MEF TTC19 +/- Cumene hydroperoxide
MEF TTC19 -/- Untreated
MEF TTC19 -/- PYO
MEF TTC19 -/- Cumene hydroperoxide
b
BODIPY Oxidized 510 nm
BODIPY Reduced 590 nm
BODIPY Oxidized 510 nm
BODIPY Reduced 590 nm
MEF TTC19 +/- Untreated
MEF TTC19 +/- 2 months PYO
MEF TTC19 +/- Cumene hydroperoxide
MEF TTC19 -/- Untreated
MEF TTC19 -/- 2 months PYO
MEF TTC19 -/- Cumene hydroperoxide

Figure S2

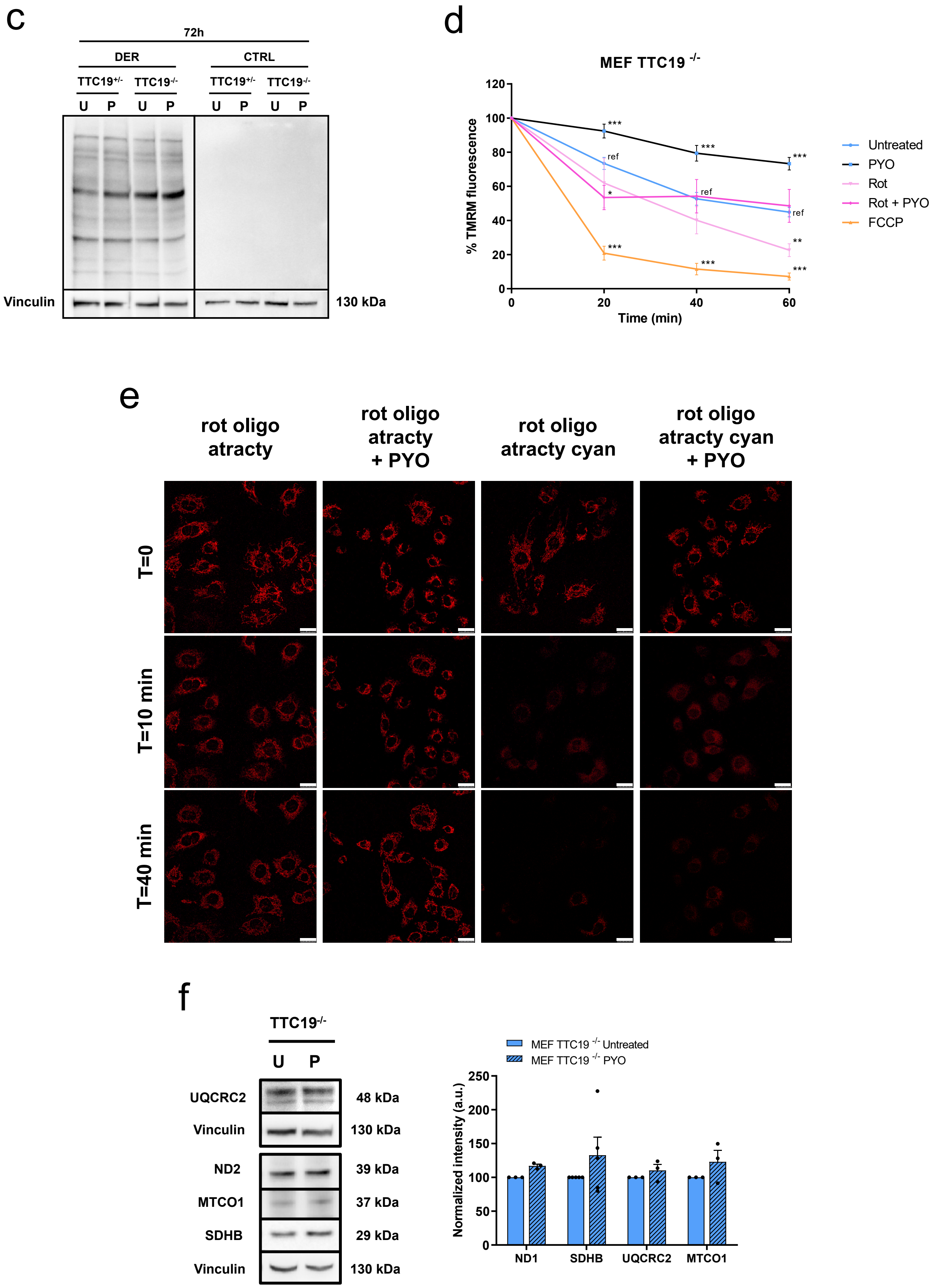

c
72h
DER
CTRL
TTC19+/-
TTC19-/-
U
P
Vinculin
130 kDa
d
MEF TTC19 -/-
% TMRM fluorescence
Time (min)
Untreated
PYO
Rot
Rot + PYO
FCCP
ref
e
rot oligo atracty
rot oligo atracty + PYO
rot oligo atracty cyan
rot oligo atracty cyan + PYO
T=0
T=10 min
T=40 min
f
TTC19-/-
U P
UQCRC2
48 kDa
Vinculin
130 kDa
ND2
39 kDa
MTCO1
37 kDa
SDHB
29 kDa
Vinculin
130 kDa
MEF TTC19 -/- Untreated
MEF TTC19 -/- PYO
Normalized intensity (a.u.)
ND1
SDHB
UQCRC2
MTCO1

Figure S2. **PYO does not oxidize MEF proteins but has a tendency to increase RC expression in MEF TTC19$^{-/-}$.**

a-b) Representative images of MEF TTC19$^{+/-}$ and MEF TTC19$^{-/-}$ either untreated or treated with 1.5 µM PYO for 72 h (a) or for 2 months (b). Cumene hydroperoxide (CH) was used as positive control. Scale bars: 50 µm. Quantifications of the images are present in Figures 2b-c. **c)** Representative Oxyblot related to the quantification presented in figure 2d. **d-e)** PYO maintains mitochondrial membrane potential when complex I (d) and complex V (e) are inhibited. In d, experiments were performed as in Figure 1h and 1i (means ± SEM, n=6 independent experiments). In e, representative images were obtained as in Figure 1g and refer to Figure 1i. Scale bars: 25µm. **f)** Representative Western blot images (on the left) and the relative quantifications (on the right) of different proteins composing respiratory chain complexes: UQCRC2 for CIII, ND2 for CI, MTCO1 for CIV, SDHB for CII. MEF TTC19$^{-/-}$ were either untreated or treated with 1.5 µM PYO for 72 h. Vinculin was used as loading control (means ± SEM, for SDHB antibody n=5 independent experiments, for the others n=3 independent experiments). For all the panels statistical significance (two-way ANOVA with Bonferroni post-test or two-tailed Student's *t*-test analysis) was determined (* = $p<0.05$; **=$p<0.01$; ***=$p<0.001$).

Figure S3

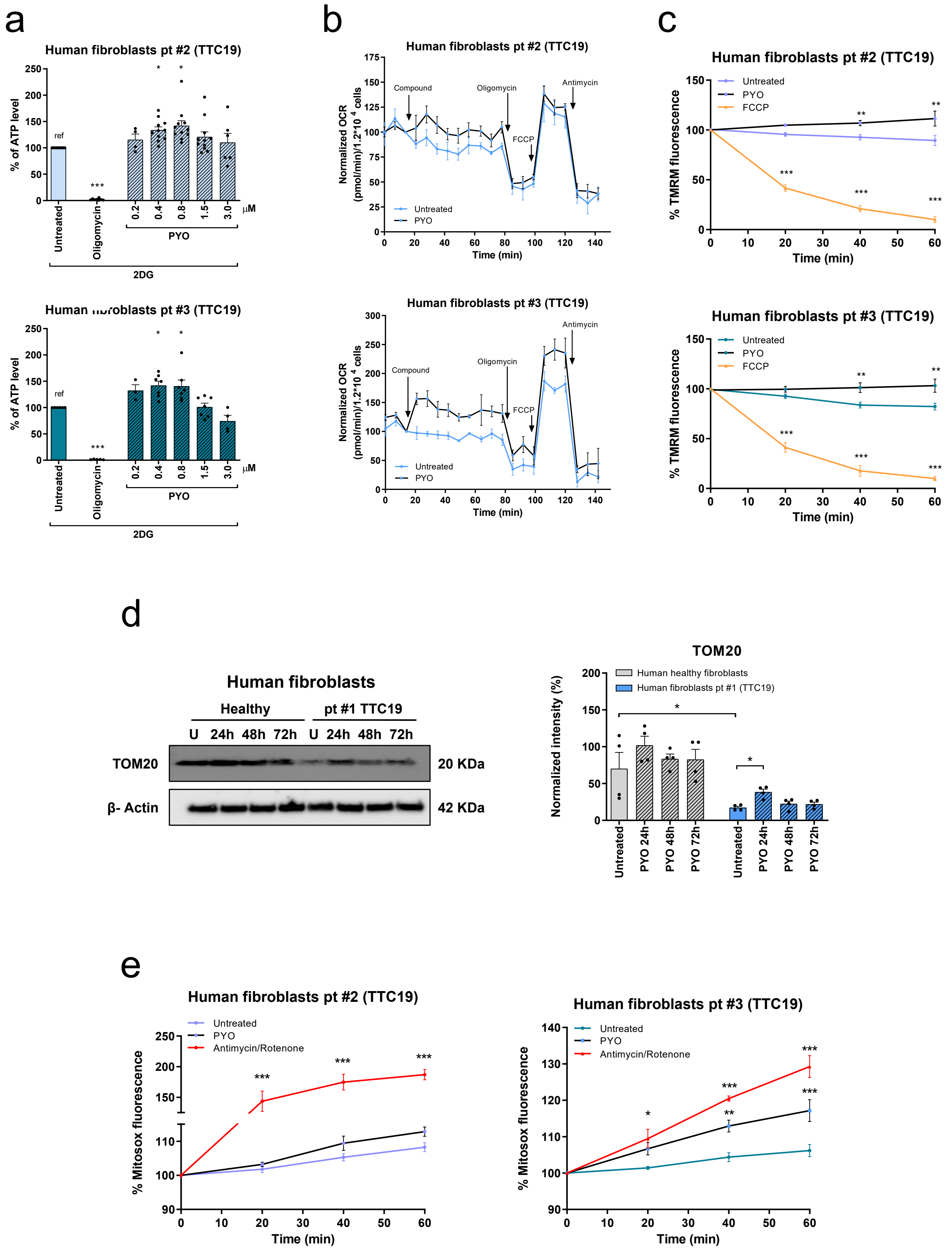
a
Human fibroblasts pt #2 (TTC19)
% of ATP level
ref
Untreated
Oligomycin
0.2
0.4
0.8
1.5
3.0
µM
PYO
2DG
Human fibroblasts pt #3 (TTC19)
b
Human fibroblasts pt #2 (TTC19)
Normalized OCR (pmol/min)/1.2*10 4 cells
Compound
Oligomycin
FCCP
Antimycin
Untreated
PYO
Time (min)
Human fibroblasts pt #3 (TTC19)
c
Human fibroblasts pt #2 (TTC19)
% TMRM fluorescence
Untreated
PYO
FCCP
Time (min)
Human fibroblasts pt #3 (TTC19)
d
Human fibroblasts
Healthy
pt #1 TTC19
U 24h 48h 72h U 24h 48h 72h
TOM20
β- Actin
20 KDa
42 KDa
TOM20
Normalized intensity (%)
Human healthy fibroblasts
Human fibroblasts pt #1 (TTC19)
Untreated
PYO 24h
PYO 48h
PYO 72h
e
Human fibroblasts pt #2 (TTC19)
% Mitosox fluorescence
Untreated
PYO
Antimycin/Rotenone
Time (min)
Human fibroblasts pt #3 (TTC19)

Figure S3

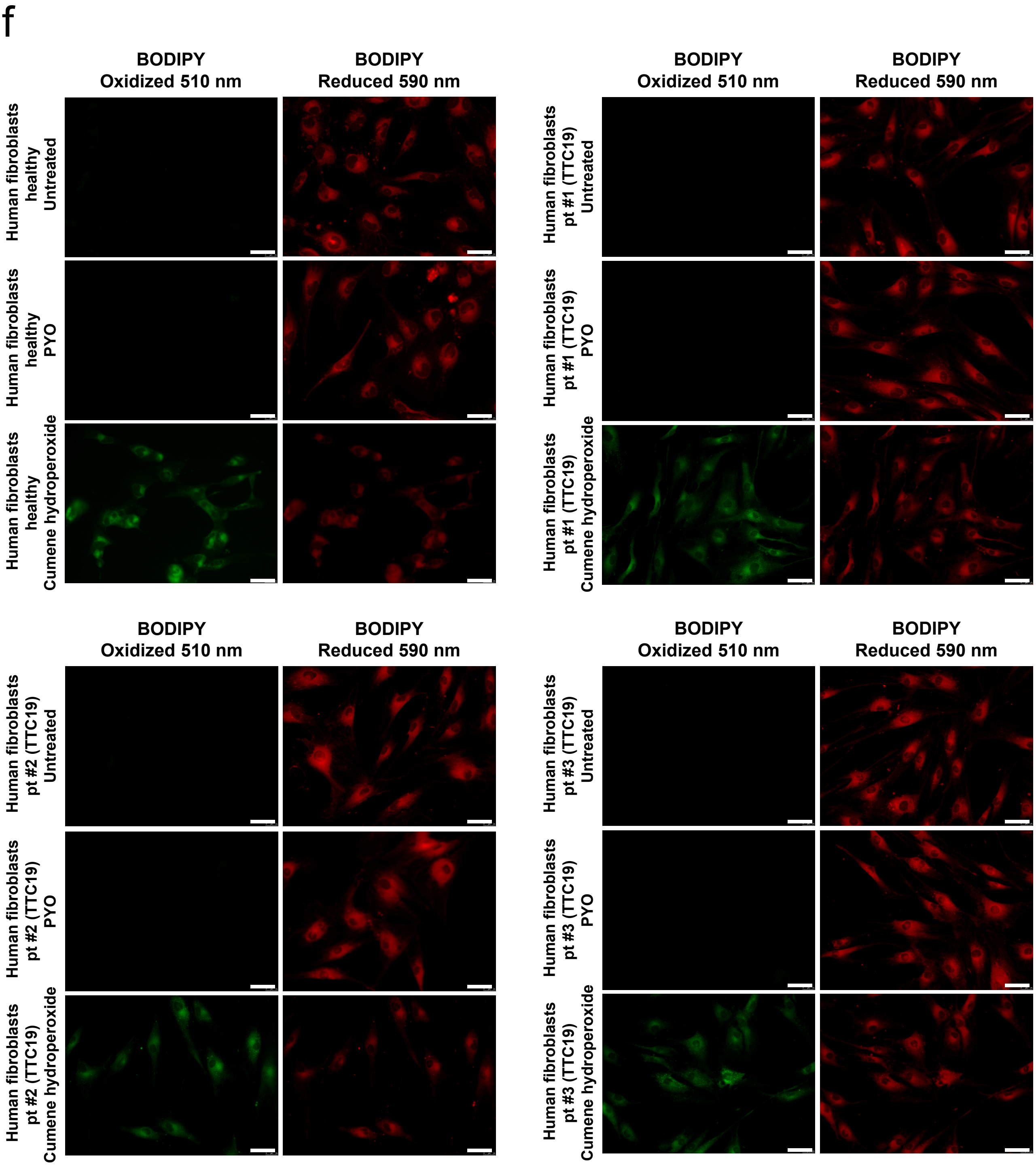
f
BODIPY
Oxidized 510 nm
BODIPY
Reduced 590 nm
Human fibroblasts
healthy
Untreated
Human fibroblasts
healthy
PYO
Human fibroblasts
healthy
Cumene hydroperoxide
Human fibroblasts
pt #1 (TTC19)
Untreated
Human fibroblasts
pt #1 (TTC19)
PYO
Human fibroblasts
pt #1 (TTC19)
Cumene hydroperoxide
Human fibroblasts
pt #2 (TTC19)
Untreated
Human fibroblasts
pt #2 (TTC19)
PYO
Human fibroblasts
pt #2 (TTC19)
Cumene hydroperoxide
Human fibroblasts
pt #3 (TTC19)
Untreated
Human fibroblasts
pt #3 (TTC19)
PYO
Human fibroblasts
pt #3 (TTC19)
Cumene hydroperoxide

Figure S3. **PYO enhances bioenergetic efficiency in fibroblasts from two other patients with *TTC19* mutation.**

a) Cellular ATP of mitochondrial origin was measured was measured in fibroblasts from patients #2 (n=11 independent assays) and #3 (n=7 independent assays) after treatment with PYO. Oligomycin was used as control. Cells were treated as in Figure 4b. Data are means ± SEM. **b)** Oxygen consumption rate (OCR) was measured in fibroblasts from patients #2 (n=4 independent experiments) and #3 (n=3 independent experiments) in the presence or absence of 0.8 μM PYO as in Figure 4d. Maximal respiration is reported in Figure 4e. Data shown are means ± SEM normalized to the untreated sample. **c)**. Mitochondrial membrane potential of fibroblasts from patients #2 (n=5 independent experiments) and #3 (n=6 independent experiments) was assessed monitoring the intensity of TMRM fluorescence after 0.8 μM PYO addition as in Figure 4g. Data shown are means ± SEM. **d)** PYO enhances the protein expression level of TOM20 in patient #1 fibroblasts. Experiments were performed as described in Figure 3d. Values are shown as means ± SEM (n=3 independent experiments). **e)** Mitochondrial ROS production by fibroblasts from patients #2 and #3 was determined by measuring the intensity of Mitosox® fluorescence as in Figure 5b. Data are means ± SEM (n=4 independent experiments). **f)** Representative fluorescence microscopy images of lipid peroxidation detection in human healthy fibroblasts and fibroblasts from patients #1, #2, #3, untreated or treated for 72 hours with 0.8 μM PYO. Cumene Hydroperoxide (CH) was used as positive control. Bars: 50 μm. Quantification is presented in Figure 5d. Statistical significance (one-way ANOVA with Dunnett's Multiple comparison test, two-way ANOVA with Bonferroni post-test, or two-tailed Student's *t*-test analysis) was determined (* = $p<0.05$; **=$p<0.01$; ***=$p<0.001$).

Figure S4

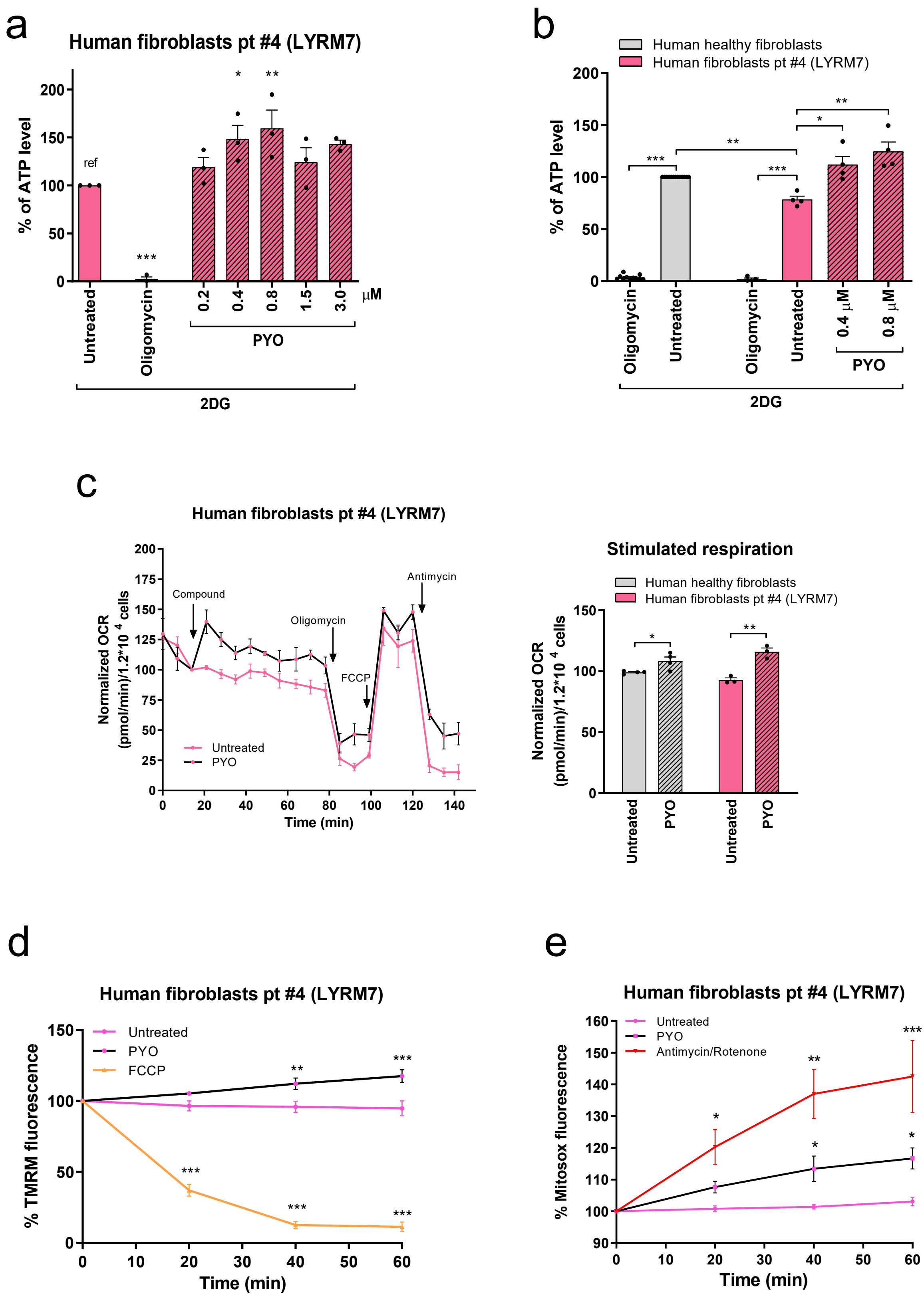
a
Human fibroblasts pt #4 (LYRM7)
% of ATP level
ref
Untreated
Oligomycin
0.2
0.4
0.8
1.5
3.0
µM
PYO
2DG
b
Human healthy fibroblasts
Human fibroblasts pt #4 (LYRM7)
% of ATP level
Oligomycin
Untreated
Oligomycin
Untreated
0.4 µM
0.8 µM
PYO
2DG
c
Human fibroblasts pt #4 (LYRM7)
Normalized OCR (pmol/min)/1.2*10 4 cells
Compound
Oligomycin
FCCP
Antimycin
Untreated
PYO
Time (min)
Stimulated respiration
Human healthy fibroblasts
Human fibroblasts pt #4 (LYRM7)
Normalized OCR (pmol/min)/1.2*10 4 cells
Untreated
PYO
Untreated
PYO
d
Human fibroblasts pt #4 (LYRM7)
% TMRM fluorescence
Untreated
PYO
FCCP
Time (min)
e
Human fibroblasts pt #4 (LYRM7)
% Mitosox fluorescence
Untreated
PYO
Antimycin/Rotenone
Time (min)

Figure S4. PYO enhances bioenergetic efficiency in fibroblast of a patient with mutation of *LYRM7*.

a-b) Cellular ATP of mitochondrial origin was measured in human healthy fibroblasts and fibroblasts from a patient harbouring a mutation in the *LYRM7* gene (human fibroblasts pt#4), with or without treatment with PYO. Oligomycin was used as control. Cells were treated for 1 h in a fresh medium, in which glucose was replaced with 5.5 mM 2-deoxyglucose (2DG), to inhibit glycolysis. In (a), values are reported as percentage of the luciferase signal with respect to the untreated sample, labeled "ref" in the figure (n=3 independent assays). In (b), values are reported as percentage of luciferase signal with reference to the untreated human healthy fibroblasts (in gray) (n=4 independent assays). Values are means ± SEM. **c)** OCR by fibroblasts from patient #4 was measured in the presence or absence of 0.8 μM PYO. Values were normalized with respect to the basal respiration recorded before compound addition, which was taken as 100%, and are the means ± SEM (n=3 independent experiments). Bioenergetic parameters were calculated as in Figure 1d. Maximal respiration is reported on the right of the figure. Values are means ± SEM with reference to the untreated sample (n=3 independent experiments). **d)** Mitochondrial membrane potential of patient #4 fibroblasts was assessed monitoring the intensity of TMRM fluorescence after 0.8 μM PYO addition as in Figure 2h. Data shown are means ± SEM (n=4 independent experiments). **e)** Mitochondrial ROS production by fibroblasts from patient #4 was monitored as in Figure S3e. Data shown are means ± SEM (n=3 independent experiments). Statistical significance (one-way ANOVA with Dunnett's Multiple comparison test, two-way ANOVA with Bonferroni post-test, or two-tailed Student's *t*-test analysis) was determined (* = $p<0.05$; **=$p<0.01$; ***=$p<0.001$).

Figure S5

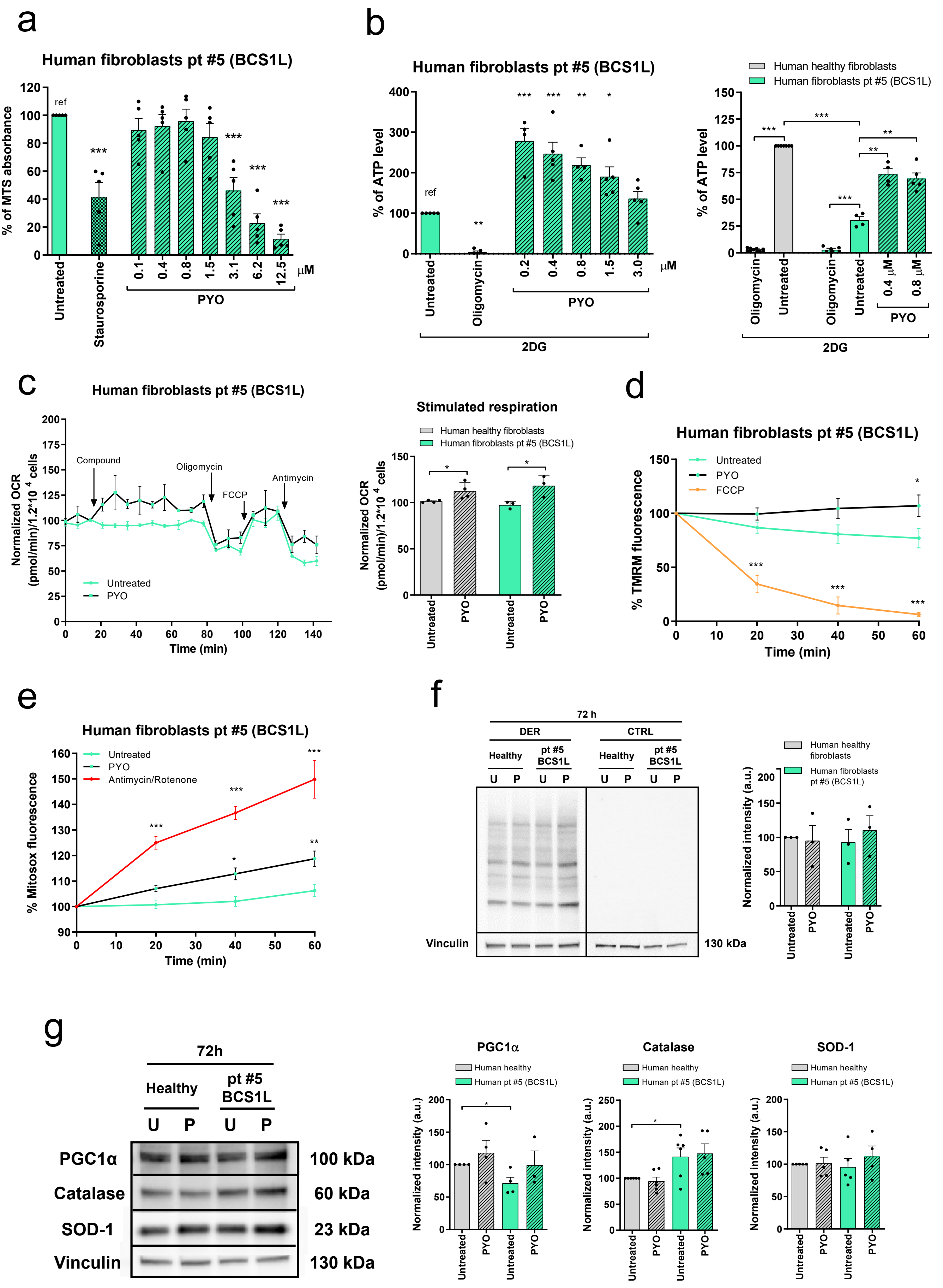
a
Human fibroblasts pt #5 (BCS1L)
% of MTS absorbance
ref
***
Untreated
Staurosporine
0.1
0.4
0.8
1.5
3.1
6.2
12.5
μM
PYO
b
Human fibroblasts pt #5 (BCS1L)
% of ATP level
ref
**
*
Untreated
Oligomycin
0.2
0.4
0.8
1.5
3.0
μM
PYO
2DG
Human healthy fibroblasts
Human fibroblasts pt #5 (BCS1L)
Oligomycin
Untreated
0.4 μM
0.8 μM
PYO
2DG
c
Human fibroblasts pt #5 (BCS1L)
Normalized OCR (pmol/min)/1.2*10 4 cells
Compound
Oligomycin
FCCP
Antimycin
Untreated
PYO
Time (min)
Stimulated respiration
Human healthy fibroblasts
Human fibroblasts pt #5 (BCS1L)
d
Human fibroblasts pt #5 (BCS1L)
% TMRM fluorescence
Untreated
PYO
FCCP
Time (min)
e
Human fibroblasts pt #5 (BCS1L)
% Mitosox fluorescence
Untreated
PYO
Antimycin/Rotenone
Time (min)
f
72 h
DER
CTRL
Healthy
pt #5 BCS1L
U
P
Vinculin
130 kDa
Human healthy fibroblasts
Human fibroblasts pt #5 (BCS1L)
Normalized intensity (a.u.)
Untreated
PYO
g
72h
Healthy
pt #5 BCS1L
U
P
PGC1α
Catalase
SOD-1
Vinculin
100 kDa
60 kDa
23 kDa
130 kDa
PGC1α
Catalase
SOD-1
Human healthy
Human pt #5 (BCS1L)
Normalized intensity (a.u.)
Untreated
PYO

Figure S5

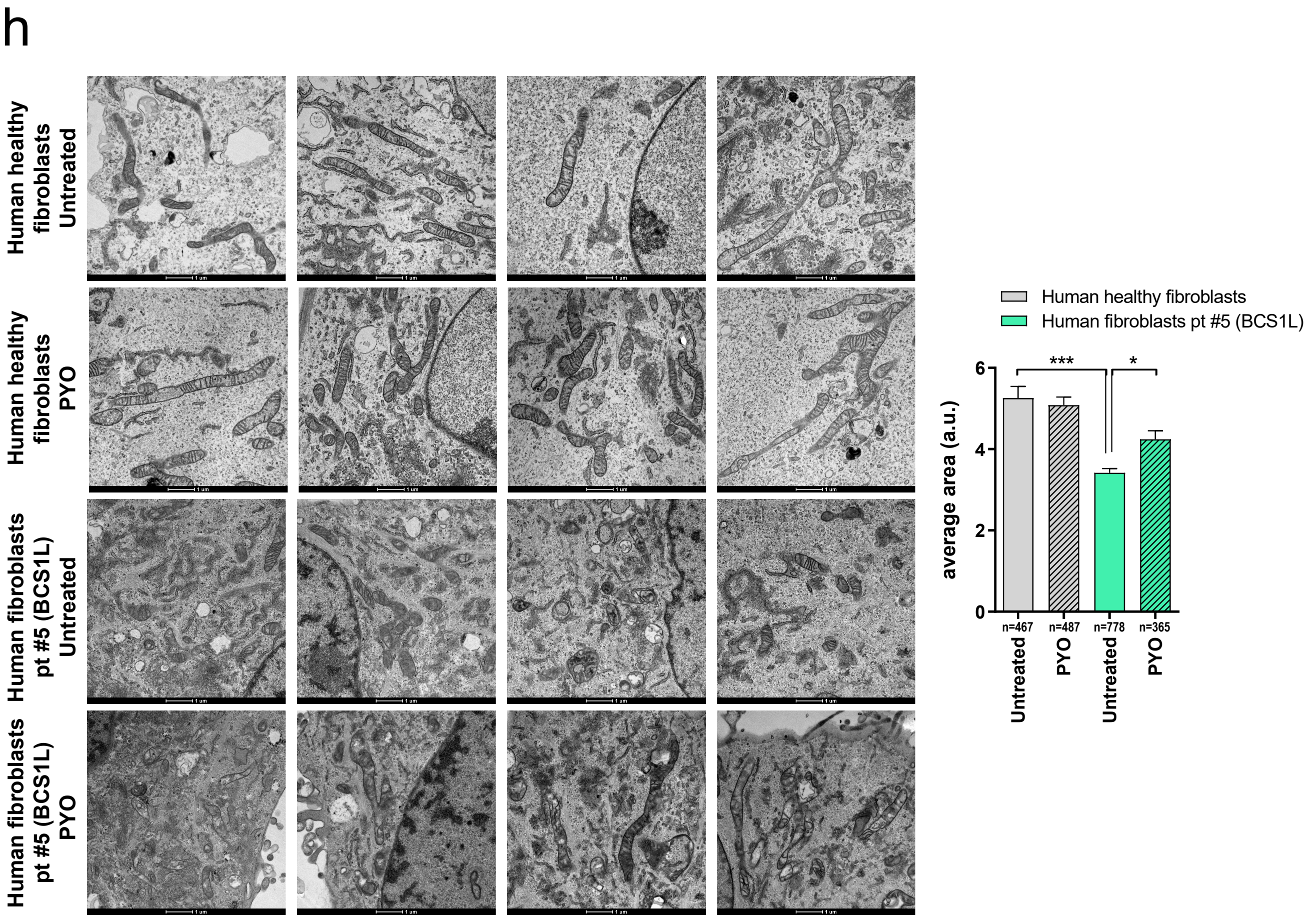
h
Human healthy fibroblasts Untreated
Human healthy fibroblasts PYO
Human fibroblasts pt #5 (BCS1L) Untreated
Human fibroblasts pt #5 (BCS1L) PYO
1 um
Human healthy fibroblasts
Human fibroblasts pt #5 (BCS1L)
average area (a.u.)
6
4
2
0
***
*
n=467
n=487
n=778
n=365
Untreated
PYO
Untreated
PYO

Figure S5. PYO recovers mitochondrial function in fibroblasts from a patient with *BCS1L* mutation.

a) Viability MTS assays were performed on fibroblasts from a patient harboring mutation in the *BCS1L* gene (human fibroblasts pt#5). Cells were either left untreated or treated with different dosages of PYO for 24 hours to determine PYO concentration that didn't affect cell survival. Positive control: 4 μM staurosporine. Data are means ± SEM of percentages of MTS absorbance (at 490 nm) with respect to the untreated sample (ref) (n=5 independent assays). **b)** Mitochondrial ATP content was measured healthy fibroblasts and fibroblasts from patient #5 as in Figure S4a-b. Values are means ± SEM (n=5 independent assays). **c)** OCR by fibroblasts from patient #5 was measured in the presence or absence of 0.8 μM PYO as in Figure S4c. Stimulated respiration were calculated as in Figure 1d. Values are means ± SEM referring to the untreated sample (n=4 independent experiments). **d)** Mitochondrial membrane potential of patient #5 fibroblasts was assessed monitoring the intensity of TMRM fluorescence after 0.8 μM PYO addition as in Figure 2h. Data shown are means ± SEM (n=4). **e)** Mitochondrial ROS production of fibroblasts from patients #5 was monitored as in Figure S3e. Data shown are means ± SEM (n=4 independent experiments). **f)** Representative Oxyblot and the quantification of the protein oxidation level in healthy fibroblasts and fibroblasts from patient #5, both untreated and treated with 0.8 μM PYO for 72 h (means ± SEM, n=3 biologically independent samples). **g)** Representative Western blot and the relative quantification of the indicated protein expression levels in healthy fibroblasts and fibroblasts from patients #5, both untreated and treated with 0.8 μM PYO for 72 h as in Figure 2f (means ± SEM, for PGC1α antibody n=4 independent experiments, for catalase antibody n=6 independent experiments, for SOD-1 antibody n=5 independent experiments). **h**) Representative transmission electron microscopy images of healthy fibroblasts and fibroblasts from patient #5, untreated or treated for 72 hours with 0.8 μM PYO. Data are means ± SEM. The number of counted mitochondria is shown in the image. Statistical significance (one-way ANOVA with Dunnett's Multiple comparison test, two-way ANOVA with Bonferroni post-test, or two-tailed Student's *t*-test) was determined for all panels (* = p<0.05; **=p<0.01; ***=p<0.001).

Figure S6

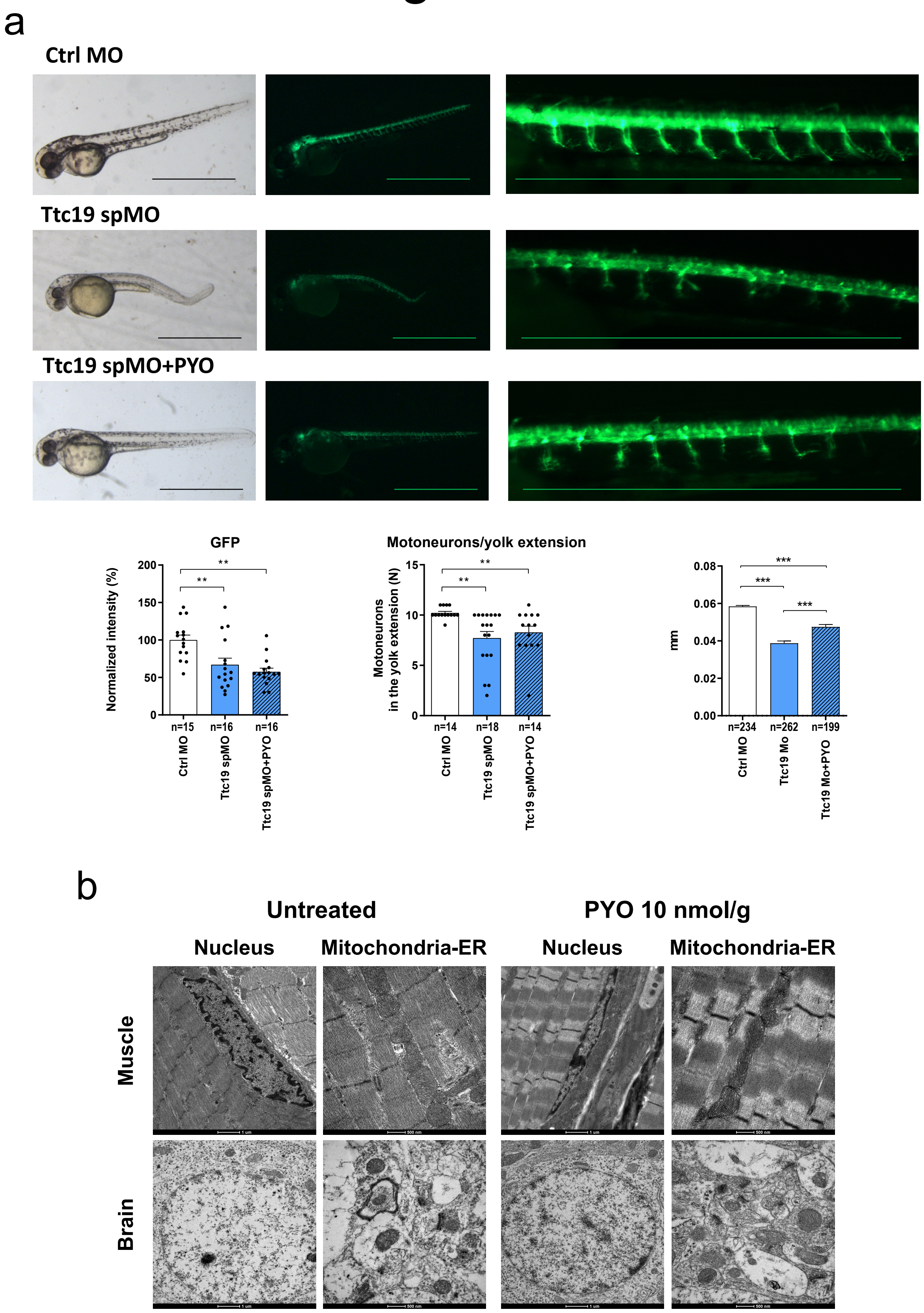

a
Ctrl MO
Ttc19 spMO
Ttc19 spMO+PYO
GFP
Normalized intensity (%)
200
150
100
50
0
**
**
n=15
n=16
n=16
Ctrl MO
Ttc19 spMO
Ttc19 spMO+PYO
Motoneurons/yolk extension
Motoneurons in the yolk extension (N)
15
10
5
0
**
**
n=14
n=18
n=14
Ctrl MO
Ttc19 spMO
Ttc19 spMO+PYO
mm
0.08
0.06
0.04
0.02
0.00
***
***
***
n=234
n=262
n=199
Ctrl MO
Ttc19 Mo
Ttc19 Mo+PYO
b
Untreated
PYO 10 nmol/g
Nucleus
Mitochondria-ER
Nucleus
Mitochondria-ER
Muscle
Brain

Figure S6. PYO affects axon development in zebrafish embryos and does not alter cellular ultrastructure in WT mice upon long-term treatment.

a) *D. Rerio* embryos were treated as in Figure 7c. The peripheral nervous system was investigated in Tg(mnx1:mGFP), which specifically highlights motoneurons in vivo. *TTC19* knock-down reduces GFP expression and the number of motoneurons in the region of the yolk extension (N/Area). PYO supplementation does not rescue those parameters, however PYO improves axons development in comparison to DMSO treated embryos. Values are means ± SEM. Quantifications of GFP expression, the number and the length of motoneuron in the region of the yolk extension are present in the figure. The numbers of the manipulated embryo and the numbers of the motoneuron counted for each condition are represented in the image. Bars: 1 mm. Statistical significance (two-tailed Student's *t*-test) was determined (*= $p<0.05$; **=$p<0.01$; ***=$p<0.001$). Bright-field images and green-fluorescent images of morphants are shown. Magnifications: 2.5X and 12 X. Bars: 1 mm. **b)** Representative TEM images of muscle and brain sections from mice untreated or treated with PYO (10 nmol/g). See also Figure 8d. Nucleus, mitochondria, ER, and the other detected organelles did not show any visible alterations. Scale bars related to Nucleus images: 1µm. Scale bars related to Mitochondria-ER images: 500 nm.

Movie 1. ***TTC19*** **KD zebrafish recover ETR after the treatment with 100 nM PYO.** It is shown that ETR is induced by a single and gentle stimulation at the tail of the larvae, which leads the embryo escaping to reach the border of 15 cm Petri disk.

Movie 2. PYO shows no toxicity in vivo in mice. The movie shows adult mice treated with PYO (10 nmol/gbw) i.p. once daily, 5 days a week, for two months. Mice are active and show no signs of distress.

Supplementary Table 1.

Primers used in the study.

Target	Sequence (From 5' to 3')
m_PGC1α (for)	GGACATGTGCAGCCAAGACTCT
m_PGC1α (rev)	CACTTCAATCCACCCAGAAAGCT
m_ Actin (for)	CTAAGGCCAACCGTGAAAAG
m_ Actin (rev)	ACCAGAGGCATACAGGGACA
d_Rp49 (for)	ATCGGTTACGGATCGAACAA
d_Rp49 (rev)	GACAATCTCCTTGCGCTTCT
d_PGC1α (for)	GGAGGAAGACGTGCCTTCTG
d_PGC1α (rev)	TACATTCGGTGCTGGTGCTT
