# Supplementary material for: Exploiting pyocyanin to treat mitochondrial disease due to respiratory complex III dysfunction
Source: Nat Commun. 2021 Apr 8;12:2103. doi: 10.1038/s41467-021-22062-x (PMC8032734; doi:10.1038/s41467-021-22062-x)
Supplement: Supplementary file 3 — Description of Additional Supplementary Files [file 41467_2021_22062_MOESM3_ESM.pdf]

Description of Additional Supplementary Files

Title: Supplementary Movie 1.

Description: TTC19 KD zebrafish recover ETR after the treatment with 100 nM PYO. It is shown that ETR is induced by a single and gentle stimulation at the tail of the larvae, which leads the embryo escaping to reach the border of 15 cm Petri disk.

Title: Supplementary Movie 2.

Description: PYO shows no toxicity in vivo in mice. The movie shows adult mice treated with PYO (10 nmol/gbw) i.p. once daily, 5 days a week, for two months. Mice are active and show no signs of distress.
