# Supplementary material for: Exploiting pyocyanin to treat mitochondrial disease due to respiratory complex III dysfunction
Source: Nat Commun. 2021 Apr 8;12:2103. doi: 10.1038/s41467-021-22062-x (PMC8032734; doi:10.1038/s41467-021-22062-x)
Supplement: Supplementary file 6 — Reporting Summary [file 41467_2021_22062_MOESM6_ESM.pdf]

nature research

Corresponding author(s): Ildiko Szabo

Last updated by author(s): Feb 20, 2021

Reporting Summary

Nature Research wishes to improve the reproducibility of the work that we publish. This form provides structure for consistency and transparency in reporting. For further information on Nature Research policies, see our Editorial Policies and the Editorial Policy Checklist.

Statistics

For all statistical analyses, confirm that the following items are present in the figure legend, table legend, main text, or Methods section.

n/a	Confirmed	
☐	☒	The exact sample size (*n*) for each experimental group/condition, given as a discrete number and unit of measurement
☐	☒	A statement on whether measurements were taken from distinct samples or whether the same sample was measured repeatedly
☐	☒	The statistical test(s) used AND whether they are one- or two-sided<br>*Only common tests should be described solely by name; describe more complex techniques in the Methods section.*
☐	☒	A description of all covariates tested
☐	☒	A description of any assumptions or corrections, such as tests of normality and adjustment for multiple comparisons
☐	☒	A full description of the statistical parameters including central tendency (e.g. means) or other basic estimates (e.g. regression coefficient) AND variation (e.g. standard deviation) or associated estimates of uncertainty (e.g. confidence intervals)
☐	☒	For null hypothesis testing, the test statistic (e.g. *F*, *t*, *r*) with confidence intervals, effect sizes, degrees of freedom and *P* value noted<br>*Give P values as exact values whenever suitable.*
☒	☐	For Bayesian analysis, information on the choice of priors and Markov chain Monte Carlo settings
☒	☐	For hierarchical and complex designs, identification of the appropriate level for tests and full reporting of outcomes
☒	☐	Estimates of effect sizes (e.g. Cohen's *d*, Pearson's *r*), indicating how they were calculated

Our web collection on statistics for biologists contains articles on many of the points above.

Software and code

Policy information about availability of computer code

Data collection	FACSCanto 11, Envision Manager 1.14 (Perkin Elmer), Chemidoc Imagelab 5.2.1, Wave 2.6.031 (Seahorse Bioscience), DATLab7 (Oroboros) , LAS AF Lite 2.6.3. , i-Control 1.8 SP1 (Tecan)
Data analysis	ImageJ 1.49v, Wave 2.6.0.31, iQiCycler5 v2.0, GraphPad Prism9, DATLab7 (Oroboros), GIMP2.0

For manuscripts utilizing custom algorithms or software that are central to the research but not yet described in published literature, software must be made available to editors and reviewers. We strongly encourage code deposition in a community repository (e.g. GitHub). See the Nature Research guidelines for submitting code & software for further information.

Data

Policy information about availability of data

All manuscripts must include a data availability statement. This statement should provide the following information, where applicable:

- Accession codes, unique identifiers, or web links for publicly available datasets
- A list of figures that have associated raw data
- A description of any restrictions on data availability

Raw data supporting the results are reported in the article as supporting file. Accession codes and other unique identifiers are detailed in the Materials and method sections.

Field-specific reporting

Please select the one below that is the best fit for your research. If you are not sure, read the appropriate sections before making your selection.

- [x] Life sciences
- [ ] Behavioural & social sciences
- [ ] Ecological, evolutionary & environmental sciences

For a reference copy of the document with all sections, see nature.com/documents/nr-reporting-summary-flat.pdf

Life sciences study design

All studies must disclose on these points even when the disclosure is negative.

Sample size	Samples size was always higher than 3 in all experiments referring to biological replicates. In many cases, technical replicates were also performed. No sample size pre-calculation was performed. The sample sizes were chosen on the basis of previously published in vitro and in vivo (Drosophila, zebrafish, mice) data with the models used in this study. The sample sizes used in this study are sufficient as they gave statictically significant results.
Data exclusions	No data were excluded. Samples size was always higher than 3 in all experiments referring to biological replicates.
Replication	All attempts at replication (at least 3 independent biological and at least 3 technical replicates) were successful.
Randomization	Mice, fishes selected randomly, for flyes only males were used as the phenotype of TTC19 KO male flyes is more severe (statistically significant difference, as published by the authors in a previous paper).
Blinding	Experiments and analysis were performed by different persons to ensure blinded experiments and evaluation.

Reporting for specific materials, systems and methods

We require information from authors about some types of materials, experimental systems and methods used in many studies. Here, indicate whether each material, system or method listed is relevant to your study. If you are not sure if a list item applies to your research, read the appropriate section before selecting a response.

Materials & experimental systems

n/a	Involved in the study	
[ ]	[x]	Antibodies
[ ]	[x]	Eukaryotic cell lines
[x]	[ ]	Palaeontology and archaeology
[ ]	[x]	Animals and other organisms
[ ]	[x]	Human research participants
[x]	[ ]	Clinical data
[x]	[ ]	Dual use research of concern

Methods

n/a	Involved in the study	
[x]	[ ]	ChIP-seq
[ ]	[x]	Flow cytometry
[x]	[ ]	MRI-based neuroimaging

Antibodies

Antibodies used	Primary antibodies: mitofusin-2 (1:1000 in 5% skim milk – Abnova, H00009927-M03, clone4H8); ATP5A (1:1000 in 5% skim milk – Abcam AB14748 [15H4C4], lot: GR209582-7); β-actin (1:3000 in TTBS - EMD Millipore, MAB1501, clone C4, lot: 3166064); PGC1α (1:1000 in TBS + 5% BSA – Abcam AB54481, lot: GR3315850-1); ND2 (1:500 in TTBS – Thermo Fisher Scientific, PA5-37185, lot: TC2524471C); SDHB (1:1000 in PBST + 3% BSA – Abcam, AB14714 [21A11AE7], lot:GR3272683-1); UQCRC2 (1:1000 in PBST + 3% BSA – Abcam, AB110252 [16D10AD9AH5], lot:GR3254174-5); MTCO1 (1:1000 in PBST + 3% BSA – Abcam, AB14705 [1D6E1A8]); SOD-1 (1:1000 in TTBS - Santa Cruz Biotechnology, sc11407 (FL-154)); CAT (1:1000 in TBS + 5% BSA – Abcam, AB1877); Vinculin (1:2000 in TTBS – Millipore, AB6039, lot: 3215996); Na+K+ATPase (1:2000 in TTBS -Abcam, AB7671, clone 464.6); Hsp70 (1:5000 in PBST – Sigma-Aldrich, H5147, clone BRM-22). Anti-mouse (dilution 1:10000 in TTBS; BioRad, #1706516, lot: L005680 B) or anti-rabbit (dilution 1:10000 in TTBS; Sera Care, 5220-0336, lot: 10283380) secondary antibodies conjugated to horseradish peroxidase (HRP) were used for proteins detection.
Validation	All data about the usage (dilution, incubation time, type of membrane) are detailed in the manuscript and validation was based on information given by the manifacturers available in the datasheets reporetd on the website

Eukaryotic cell lines

Policy information about cell lines

Cell line source(s)	Human cells were obtained from the Biobank at Besta Institute, Milan, Italy.

Authentication	Cell line authetication is available at the Biobank. We identified the genetic mutations by sequencing (WES or targeted sequencing) in DNA from blood. In fibroblasts we just confirmed the presence of the identified mutation, by using PCR assays with specific primers; given the extremely low frequency of these variants, the presence of the mutation can be considered a proof that the cells derived from the patient. Additional experimental findings (i.e. absence of the protein by WB, biochemical defect of cIII) support this conclusion. All the cell lines were tested for mycoplasma contamination, and were negative. Mouse lines were obtained by the authors and described previously.
Mycoplasma contamination	All cell lines used tested negative for mycoplasma contamination.
Commonly misidentified lines (See ICLAC register)	None.

Animals and other organisms

Policy information about studies involving animals; ARRIVE guidelines recommended for reporting animal research

Laboratory animals	C57BL6/J (males and females) were used. WT zebrafish wish used and microinjection was performed on randomly separated sibling embryos at 1 cell stage. dTTC19 KO Drosophila mutant line was generated by deleting the entire CDS of the CG15173 gene using the CRISPR/Cas9 system (WellGenetics, Taipei City, Taiwan). The w1118 injection strain was used as control. Detailed information is included in the paper.
Wild animals	None.
Field-collected samples	None.
Ethics oversight	Local Ethic Committee OPBA (Organismo preposto al benessere animale) at University of Padova and Italian Ministry for Health . Experiments were carried out with the supervision of the Central Veterinary Service of the University of Padova (in compliance with Italian Law DL 116/92, embodying UE directive 86/609). Zebrafish manipulation procedures were conducted according to the Local Ethical Committee at the University of Padua and National Agency, and with the supervision of the Central Veterinary Service of the University of Padova (in compliance with Italian Law DL 116/92 and further modifications, embodying UE directive 86/609).

Note that full information on the approval of the study protocol must also be provided in the manuscript.

Human research participants

Policy information about studies involving human research participants

Population characteristics	Fibroblasts from patients with mutations in TTC19, LYRM7 or BSC1L were obtained as inidcated in Table I.
Recruitment	Subjects were recruited on the basis of harbouring mutations in TTC19, LYRM7 or BSC1L genes.
Ethics oversight	Informed consent for participation in this study was obtained from all investigated subjects in agreement with the Declaration of Helsinki. The experiments were approved by the ethical committee of the IRCCS Foundation Neurological Institute "C. Besta", Milan, Italy.

Note that full information on the approval of the study protocol must also be provided in the manuscript.

Flow Cytometry

Plots

Confirm that:

- [x] The axis labels state the marker and fluorochrome used (e.g. CD4-FITC).
- [x] The axis scales are clearly visible. Include numbers along axes only for bottom left plot of group (a 'group' is an analysis of identical markers).
- [ ] All plots are contour plots with outliers or pseudocolor plots.
- [x] A numerical value for number of cells or percentage (with statistics) is provided.

Methodology

Sample preparation	Samples were prepared after incubation with TMRM or Mitosox of the cells.
Instrument	Samples were analyzed by a FACScanto II.
Software	BD FACSDiva TM Software

Cell population abundance	10 000 cells/sample.
Gating strategy	Only debris was eliminated from analysis. Representative image for gating strategy is included in the raw data file.

☒ Tick this box to confirm that a figure exemplifying the gating strategy is provided in the Supplementary Information.
